# On single and multiple models of protein families for the detection of remote sequence relationships

**DOI:** 10.1186/1471-2105-7-48

**Published:** 2006-01-31

**Authors:** James A Casbon, Mansoor AS Saqi

**Affiliations:** 1 Bioinformatics Group, Institute of Cell and Molecular Science, The Genome Centre, Queen Mary's School of Medicine and Dentistry, Charterhouse Square, London, EC1M 6BQ, UK

## Abstract

**Background:**

The detection of relationships between a protein sequence of unknown function and a sequence whose function has been characterised enables the transfer of functional annotation. However in many cases these relationships can not be identified easily from direct comparison of the two sequences. Methods which compare sequence profiles have been shown to improve the detection of these remote sequence relationships. However, the best method for building a profile of a known set of sequences has not been established. Here we examine how the type of profile built affects its performance, both in detecting remote homologs and in the resulting alignment accuracy. In particular, we consider whether it is better to model a protein superfamily using a single structure-based alignment that is representative of all known cases of the superfamily, or to use multiple sequence-based profiles each representing an individual member of the superfamily.

**Results:**

Using profile-profile methods for remote homolog detection we benchmark the performance of single structure-based superfamily models and multiple domain models. On average, over all superfamilies, using a truncated receiver operator characteristic (*ROC*_5_) we find that multiple domain models outperform single superfamily models, except at low error rates where the two models behave in a similar way. However there is a wide range of performance depending on the superfamily. For 12% of all superfamilies the *ROC*_5 _value for superfamily models is greater than 0.2 above the domain models and for 10% of superfamilies the domain models show a similar improvement in performance over the superfamily models.

**Conclusion:**

Using a sensitive profile-profile method we have investigated the performance of single structure-based models and multiple sequence models (domain models) in detecting remote superfamily members. We find that overall, multiple models perform better in recognition although single structure-based models display better alignment accuracy.

## Background

Annotation of gene products for newly sequenced genomes is usually done electronically by transfer of functional information from proteins that have very similar amino acid sequences. However, for many of the proteins in a newly sequenced genome, a database search will not reveal a sequence which shares a high degree of sequence identity of known function and therefore no functional information can reliably be transferred. As a result many sequences are annotated as 'hypothetical protein' or 'protein of unknown function'. Typically some 30–40% of proteins in genomes sequenced so far have no annotation and this is an impediment to the exploitation of genome sequence data. Part of the difficult in inferring function from sequence is that sequence similarity is in general a sufficient but not necessary condition for functional or structural similarity and many proteins that have little discernible similarity at the sequence level have similar structures and functions. A major challenge for *in silico *annotation methods is to identify these remote relationships. Accurate identification would enable a larger proportion of the currently sequenced genomes to have putative functional annotation.

Early database searching methods compared the unknown query sequence with each database sequence in turn. More sensitive methods exploited patterns of conservation that are revealed through multiple sequence alignments by performing sequence-profile comparisons. This is, in effect the approach of intermediate searching methods and also programs such as PSI-BLAST [1,2]. More recently this approach has been extended to profile-profile comparisons [3-7].

One of the problems with constructing profiles is how to include a large number of diverse sequences: ideally one would like to include a large amount of diversity, but as more diverse sequences are included the profile is likely to be corrupted due to alignment errors. High throughput structural determination projects are generating large numbers of protein 3-dimensional structures [8,9]. Structure based multiple alignments of proteins are likely to be considerably more accurate than sequence based alignments and we would expect the corresponding profiles to be of higher quality [10-12].

Building a profile for a query sequence of unknown structure is generally done through iterative database search, as implemented in PSI-BLAST. For such sequences of unknown structure there is little choice of method since there is no structural information available. However, it is not clear what the best method is for building profiles of those proteins of known structure and different groups have therefore used differing strategies.

One approach is to build one profile representing an entire group of related proteins (a protein superfamily). This can be done by either using a sequence alignment of the proteins, or using a structure-based alignment of the proteins depending on the availability of sufficient number of 3-D structures for members of the superfamily. The superfamily model can be enriched with close hits from sequence databases to the proteins being modelled, and hybrid profiles with secondary structural information included have shown added value [5]. The alternative strategy is to build individual sequence profiles for each protein in the family, the strategy we refer to as domain models. This is the strategy used by Gough [13].

Does a single superfamily model of a large number of diverse sequences perform better at the detection of remote superfamily members than using multiple domain models built for each individual member of the superfamily? Gough et al concluded that multiple models were more effective [13].

We feel that the question of whether to build domain or superfamily models to represent a superfamily is worth revisiting for a number of reasons. Firstly, recent years have seen the development of profile/profile comparison methods. Secondly, Gough et al only tested how many hits were returned beneath a threshold score. In this paper, we use ROC analysis to examine how many hits are returned from *all possible *true relationships, where true relationships are defined by SCOP superfamilies. The SCOP database uses structure to group related proteins, and therefore some of these relationships would not be apparent from sequence considerations alone [14]. Finally, we also examine the alignment accuracy produced by the differing models, a question not addressed by Gough et al.

## Results

### Remote homolog detection

**Figure 1:**
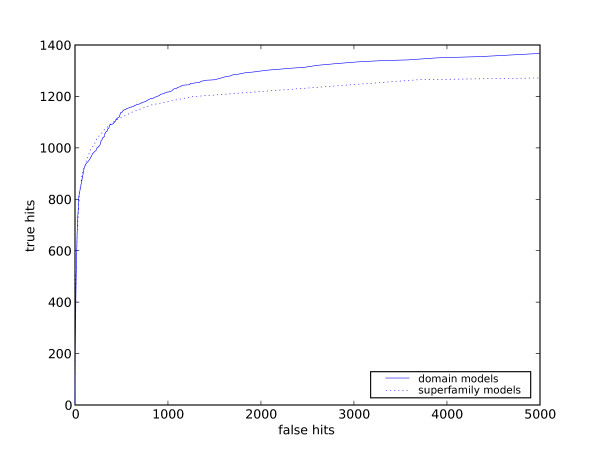
**ROC curves for superfamily and domain models**. ROC curves showing number of true positives against false positives for both types of models on the test dataset.

Figure 1 shows the ROC curves for all the data for both domain and superfamily models. The area under the curve for the domain models is much larger and, in addition, more remote homologs are detected overall (around 9% more of all possible true hits). This indicates that domain models are better at detecting remote homologs.

**Figure 2:**
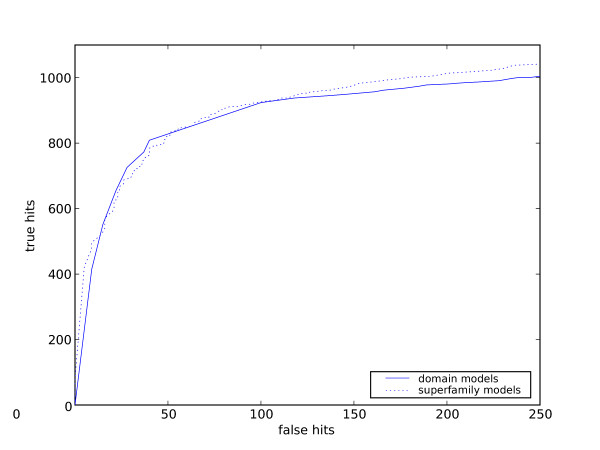
**ROC curves for superfamily and domain models at low error rates**. The same curves as figure 1, but for lower error rates.

However, in practice, when annotating, one only wishes to consider the region of reliable matches. There are approximately 250,000 potential false hits in the database. An error rate (percent of possible errors seen, not percentage of errors in hits) of 0.1% corresponds to 250 hits. Figure 2 shows the same ROC curves, but in this region of much lower probability of error. In this plot, the superfamily models have a slightly larger area under the curve. They also detect up to 5% more true hits for the same number of errors as the domain models.

### Superfamily specific truncated ROC analysis

**Figure 3:**
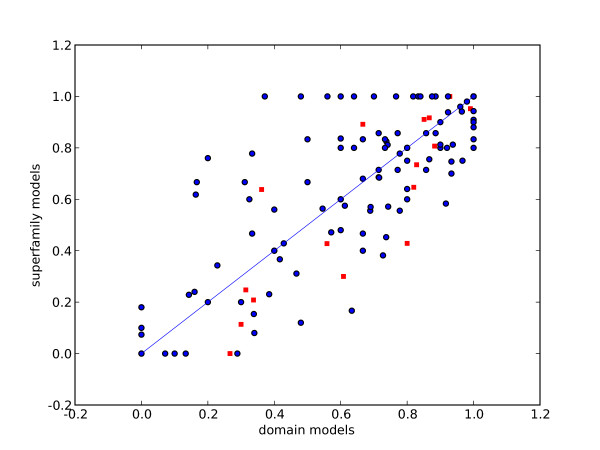
*ROC*_*n *_**values for each superfamily**, *n *= 5. Truncated ROC values for each superfamily, domain models against superfamily models. Squares show populous superfamilies as listed in table 1.

Figure 3 shows the truncated *ROC*_*n *_(*n *= 5) values of superfamily models against domain models, where each point is specific to queries from the same superfamily. In general the performance of both types of models for each superfamily is related, confirmed by the correlation coefficient of 0.7. Nevertheless, there are a number of superfamilies where performance is much better for either type of model. There are 18 (12% of all) superfamilies where the *ROC*_5 _value for superfamily models is greater than 0.2 above the domain models, corresponding to detection of 20% more homolgous relationships. Conversely, there 15 (10%) superfamilies where domain models detect the same number more than superfamily models.

The sixteen superfamilies in our dataset with more than 20 domains are also shown in the Figure 3. These represent large and sequence diverse superfamilies (see table 1). A number of these large, diverse superfamilies such as the S-adenosyl methyltransferases, alpha-beta hydrolases, cytochrome c, thioredoxin and Immunoglobulin perform well with both domain and superfamily models, with *ROC*_5 _values greater than 0.8. Similarly the 'winged-helix' DNA binding domain, the 4-helical cytokines, the nucleic-acid binding domain and the E-set domain perform poorly with both models. For a few superfamilies there exists a large difference in performance between the single and multiple models: the FAD/NAD(P) superfamily performs better with the superfamily model than with the domain models. Conversely, the NAD(P) superfamily performs better with the domain models.

**Table 1:** The 16 superfamilies in the dataset with more than 20 domains and their unique identifiers (sunids).

(Trans)glycosidases	51445
4-helical cytokines	47266
alpha/beta-Hydrolases	53474
Cytochrome c	46626
E Set domains	81296
FAD/NAD(P)-binding	51905
Fibronectin type	49265
Homeodomain-like	46689
Immunoglobulin	48726
NAD(P)-binding	51735
Nucleic acid-binding	50249
P-loop	52540
S-adenosyl	53335
Thioredoxin-like	52833
Viral coat	49611
Winged helix	49625

### Alignment accuracy

**Figure 4:**
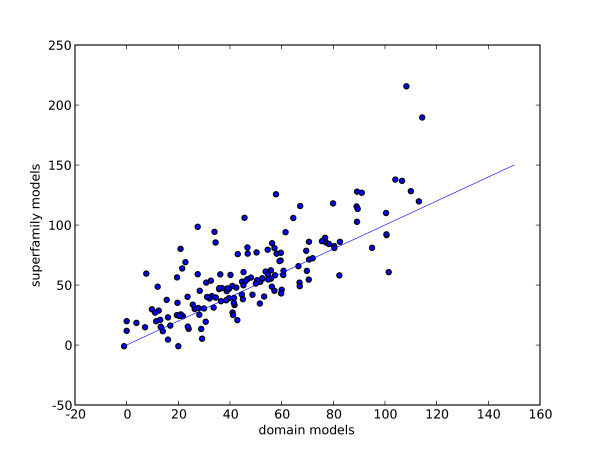
**average number of correctly aligned positions for both types of model**. Each dot shows the average number of aligned positions across each superfamily for superfamily models against domain models.

Figure 4 shows the average alignment accuracy of each superfamily using the two types of models. The figure shows that for most superfamilies, the superfamily models align more positions correctly than the domain models. Linear regression shows a slope of 1.04 and y-intercept of 8.61, r^2 ^= 0.63. This indicates that, in general, on average for a superfamily, we can expect around 8 more residues to be aligned correctly that for domain models.

## Discussion

Does a single profile of a protein superfamily built from *structure*-*based *alignments perform better at recognition than multiple domain models? The comparison is not straightforward and this analysis identifies some of the factors that are important in a comparison of single and multiple models using profile-profile methods.

The structure-based multiple alignments used to build the profiles for single models may be poor for some of the superfamilies, although in the absence of suitable expert reference alignments this is difficult to assess. A detailed assessment of the validity of the alignment method is described in [12]. In addition, the definition of a superfamily is not without limitations and may change.

Globally, the use of a set of models representing domains is preferable to using superfamily models. This is in line with previous results ([13]). However, for low error thresholds, both types of models perform similarly in terms of the number of homolgous relationships detected. In terms of particular superfamilies, the situation is different. Over 20% of the superfamilies tested there was a large difference in performance of domain or superfamily models, evenly distributed to favour either model.

In addition to the ability to detect homologs, producing an accurate alignment is also important. We have investigated the accuracy of the alignments produced by both types of model. For many superfamilies, the superfamily model correctly aligns more positions. This suggests that examination of the scoring scheme used for superfamily models could be improved, thereby increasing the accuracy of homology detection.

## Conclusion

Using a sensitive profile-profile method we have investigated the performance of single structure-based models and multiple sequence models (domain models) in detecting remote superfamily members. We find that overall, multiple models perform better in recognition although single structure-based models display better alignment accuracy.

## Methods

### Dataset

SCOP version 1.63 was used, and from this ASTRAL was used to select all sequences with less than ten percent sequence identity. From this set, all superfamilies with five or more domains were selected using the SCOP modules from Biopython [15]. The result was a set of 1718 domains distributed over 149 superfamilies.

### Profile generation

#### Domain models

For each domain in the dataset, *d*, a five round PSI-BLAST search was carried out against the UniRef50 database [2,16]. From all the sequences returned, a multiple alignment was created using the sequences with an e-value of less than 0.0005. The resulting multiple alignment was then turned into a hidden Markov model representing *D *using the program HHmake [7]. The HMM is termed *h*_*d*_.

#### Superfamily models

For each superfamily in the dataset *s*, a single structure-based multiple alignment of the corresponding domains in *s *was produced according to the same protocol as the S4 database [12]. The resulting multiple alignment is used to generate an HMM of *s*, *h*_*s*_.

### Profile searching

The program HHsearch was used to search databases of HMMs [7]. HHsearch searches a database of HMMs and reports hits and the alignments of the query model to the hit. HHsearch was run using the "-p 0" option to report the score of all hits with a probability greater than zero.

### Assessing performance

#### Homology recognition

To quantify the performance of a the domain and superfamily models, a leave-one-out test was performed. In turn, a model of each leave-one-out domain hdx
 MathType@MTEF@5@5@+=feaafiart1ev1aaatCvAUfKttLearuWrP9MDH5MBPbIqV92AaeXatLxBI9gBaebbnrfifHhDYfgasaacH8akY=wiFfYdH8Gipec8Eeeu0xXdbba9frFj0=OqFfea0dXdd9vqai=hGuQ8kuc9pgc9s8qqaq=dirpe0xb9q8qiLsFr0=vr0=vr0dc8meaabaqaciaacaGaaeqabaqabeGadaaakeaacqWGObaAdaWgaaWcbaGaemizaq2aaSbaaWqaaiabdIha4bqabaaaleqaaaaa@3133@ was searched against two databases.

The first database is a database of all domain models except the test domain models, ∪hdi
 MathType@MTEF@5@5@+=feaafiart1ev1aaatCvAUfKttLearuWrP9MDH5MBPbIqV92AaeXatLxBI9gBaebbnrfifHhDYfgasaacH8akY=wiFfYdH8Gipec8Eeeu0xXdbba9frFj0=OqFfea0dXdd9vqai=hGuQ8kuc9pgc9s8qqaq=dirpe0xb9q8qiLsFr0=vr0=vr0dc8meaabaqaciaacaGaaeqabaqabeGadaaakeaacqGHQicYcqWGObaAdaWgaaWcbaGaemizaq2aaSbaaWqaaiabdMgaPbqabaaaleqaaaaa@32B5@, *i *≠ *x*. The second database is a database of all superfamily models, ∪dsi
 MathType@MTEF@5@5@+=feaafiart1ev1aaatCvAUfKttLearuWrP9MDH5MBPbIqV92AaeXatLxBI9gBaebbnrfifHhDYfgasaacH8akY=wiFfYdH8Gipec8Eeeu0xXdbba9frFj0=OqFfea0dXdd9vqai=hGuQ8kuc9pgc9s8qqaq=dirpe0xb9q8qiLsFr0=vr0=vr0dc8meaabaqaciaacaGaaeqabaqabeGadaaakeaacqGHQicYcqWGKbazdaWgaaWcbaGaem4Cam3aaSbaaWqaaiabdMgaPbqabaaaleqaaaaa@32CB@. The single model corresponding to the same superfamily as *d*_*x *_is altered to remove *d*_*x*_, to remove any information from the query domain.

The result of a search against the superfamily model database will be a list of expect values for esi
 MathType@MTEF@5@5@+=feaafiart1ev1aaatCvAUfKttLearuWrP9MDH5MBPbIqV92AaeXatLxBI9gBaebbnrfifHhDYfgasaacH8akY=wiFfYdH8Gipec8Eeeu0xXdbba9frFj0=OqFfea0dXdd9vqai=hGuQ8kuc9pgc9s8qqaq=dirpe0xb9q8qiLsFr0=vr0=vr0dc8meaabaqaciaacaGaaeqabaqabeGadaaakeaacqWGLbqzdaWgaaWcbaGaem4Cam3aaSbaaWqaaiabdMgaPbqabaaaleqaaaaa@312D@ all superfamilies *s*_*i *_in the database. For the domain model database, the result will be a list of expect values edi
 MathType@MTEF@5@5@+=feaafiart1ev1aaatCvAUfKttLearuWrP9MDH5MBPbIqV92AaeXatLxBI9gBaebbnrfifHhDYfgasaacH8akY=wiFfYdH8Gipec8Eeeu0xXdbba9frFj0=OqFfea0dXdd9vqai=hGuQ8kuc9pgc9s8qqaq=dirpe0xb9q8qiLsFr0=vr0=vr0dc8meaabaqaciaacaGaaeqabaqabeGadaaakeaacqWGLbqzdaWgaaWcbaGaemizaq2aaSbaaWqaaiabdMgaPbqabaaaleqaaaaa@310F@ for all domain models *d*_*i *_in the database. However, we wish to perform ROC analysis to quantify the accuracy of the search. In the domain model case, to annotate the unknown domain as belonging to a given superfamily, clearly it needs to show similarity to only one and not all members of the superfamily. Therefore, the hit list for a given query is modified by taking *e*_*s *_= min_*d*∈*s *_*e*_*d *_to give a list of e-values relating the query to superfamilies.

All the hit lists over all queries are merged to give two lists: one of (minimum e-value) hits to the domain models and one of hits to the single models. Each list is sorted by e-value and then classified as true if the hit is the same superfamily as the query, or false if it is from a different superfamily. A conventional ROC analysis can then be generated from this data.

In addition, we wish to calculate superfamily specific ROC values, to examine how the performance varies between homologous superfamilies. To calculate a superfamily specific performance for superfamily *s*, each hit list is filtered such that only queries from superfamily s remain. On each list we calculate the truncated *ROC*_*n *_value (n = 5), given by

ROCn=∑i=1nti/nT
 MathType@MTEF@5@5@+=feaafiart1ev1aaatCvAUfKttLearuWrP9MDH5MBPbIqV92AaeXatLxBI9gBaebbnrfifHhDYfgasaacH8akY=wiFfYdH8Gipec8Eeeu0xXdbba9frFj0=OqFfea0dXdd9vqai=hGuQ8kuc9pgc9s8qqaq=dirpe0xb9q8qiLsFr0=vr0=vr0dc8meaabaqaciaacaGaaeqabaqabeGadaaakeaacqWGsbGucqWGpbWtcqWGdbWqdaWgaaWcbaGaemOBa4gabeaakiabg2da9maaqahabaGaemiDaq3aaSbaaSqaaiabdMgaPbqabaGccqGGVaWlcqWGUbGBcqWGubavaSqaaiabdMgaPjabg2da9iabigdaXaqaaiabd6gaUbqdcqGHris5aaaa@4026@

where *t*_*i *_is the number of true hits before the *i*th false hit, and *T *is the total number of true hits possible.

#### Alignment accuracy

To assess the alignment accuracy of domain models, the profile alignment reported by HHsearch was compared to the structural alignment produced by SAP. If two residues equivalenced by SAP were also equivalenced by HHsearch this increased the accuracy of the alignment by one.

For superfamily models, the HHsearch alignment was compared to the S4 alignment of the superfamily. Again, for each residue correctly placed by HHsearch the accuracy was increased by one. One may object that the superfamily alignment should be recalculated without the test domain to start with rather than simply deleting the test domain. However, investigating the stability of the alignments suggests the alignments are stable to removal of one domain (see appendix A). Using the alignment with the domain removed allows calculation of the alignment accuracy.

To estimate the accuracy for a particular superfamily, the average alignment accuracy was taken over all domains in the superfamily.

## Authors' contributions

JC wrote the code, contributed to the design of the study and helped to prepare the manuscript. MASS contributed to the design of the study, helped to prepare the manuscript and provided overall project coordination. All authors read and approved the final manuscript.

## Appendix A – stability of structural alignments

We calculated how the alignments changed in order to assess whether they are stable to the removal one domain. For each domain in each superfamily, the structural alignment was generated without any information from the missing domain. We then calculated three measures of conservation:

**Correct positions: **the percentage of columns in the multiple alignment that are identical to equivalent columns in the reference alignment

**Conserved pairings: **for each position in the reference alignment with say *n *residues, we check what proportion of the *n*(*n *- 1)/2 pairings specified by the position are preserved in the test alignment.

This is averaged over all positions in the test alignment.

**Average shift: **for each *n*(*n*- 1)/2 residue pairings in each position we calculate the average shift between equivalenced residues in the test alignment.

These measures were calculated for all positions where gap content was less than 10% and averaged across each test alignment. The results are shown in figure 4.

The figure shows that the number of conserved pairings is high, typically 80–90%. However, conserved positions vary a lot. This is because some superfamilies have a larger number of sequences; given the same level of internal consistency with regard to pairings, an alignment with more sequences has a higher likelihood of error at each position. In general, the shift scores are also very low. In conclusion, it seems the alignments are stable to regeneration without one domain.

**Figure 5:**
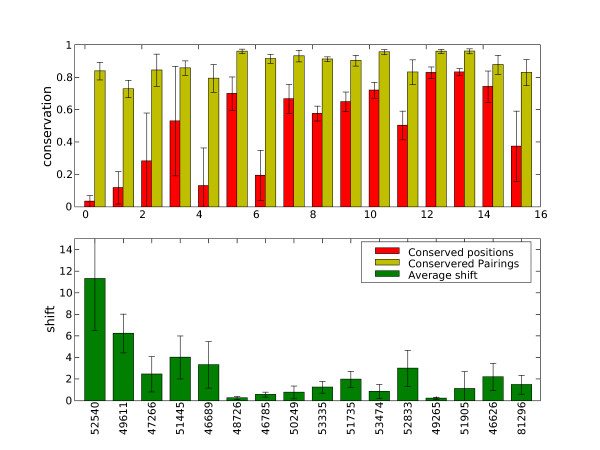
**Conservation measures for stability of alignments for each superfamily**. Error bars who one standard deviation.
